# Low-Grade Inflammation and Ultra-Processed Foods Consumption: A Review

**DOI:** 10.3390/nu15061546

**Published:** 2023-03-22

**Authors:** Marta Tristan Asensi, Antonia Napoletano, Francesco Sofi, Monica Dinu

**Affiliations:** 1 Department of Experimental and Clinical Medicine, University of Florence, 50134 Florence, Italy; 2 Unit of Clinical Nutrition, Careggi University Hospital, 50134 Florence, Italy

**Keywords:** ultra-processed foods, NOVA classification, low-grade inflammation, chronic diseases

## Abstract

Low-grade inflammation alters the homeostasis of the organism and favors the onset of many chronic diseases. The global growth in the prevalence of noncommunicable diseases in recent years has been accompanied by an increase in the consumption of ultra-processed foods (UPF). Known to be hyperpalatable, economic and ready-to-eat, increased consumption of UPF has already been recognized as a risk factor for several chronic diseases. Different research groups have tried to investigate whether UPF consumption could promote low-grade inflammation and thus favor the development of noncommunicable diseases. Current evidence highlights the adverse health effects of UPF characteristics, not only due to the nutrients provided by a diet rich in UPF, but also due to the non-nutritive components present in UPF and the effect they may have on gut health. This review aims to summarize the available evidence on the possible relationship between excessive UPF consumption and modulation of low-grade inflammation, as potential promoters of chronic disease.

## 1. Introduction

Inflammation is an immunosurveillance response essential for host defense, which serves to repair damaged tissues and eliminate toxic agents [1]. However, when this response becomes chronic, it results in the presence of immune system cells for an increasing period of time. This state of low-grade inflammation can lead to dysmetabolic conditions that disrupt homeostasis, favoring the development of a wide range of noncommunicable diseases such as cancer, diabetes and cardiovascular diseases [2].

Current evidence highlights diet among the modifiable behavioral risk factors for the development of noncommunicable diseases [3]. In recent years, particular attention has been paid to the increased consumption of ultra-processed foods (UPF) worldwide [4]. Characterized by being hyperpalatable, affordable and ready-to-eat, UPF have led to a worsening of the diet quality due to their nutritional composition [5] and have already been recognized as a risk factor for diet-related diseases [6].

Recent scientific research has sought to investigate whether UPF consumption could promote low-grade inflammation and thus favor the development of noncommunicable diseases. Emerging evidence attributes the negative effects of UPF consumption not only to the nutrients provided by a diet rich in UPF, but also to the non-nutritive components and the effect they may have on the gut microbiota. This review aims to summarize the available evidence on the possible relationship between excessive UPF consumption and modulation of low-grade inflammation as potential promoters of chronic diseases.

## 2. Low-Grade Inflammation

The inflammatory response is a defense mechanism of the innate immune system [7] that protects the host from harmful stimuli such as viruses, bacteria, toxins and infections by eliminating pathogens and promoting the repair of damaged tissues [1]. At the onset of inflammation, the innate immune cells perceive pathogen invasion or cell damage and initiate the inflammatory cascade by actively releasing soluble proinflammatory mediators. These signals also activate leukocytes and microvascular changes, such as increased vasodilation and vascular permeability, allowing leukocytes to reach the affected tissues from the blood [8]. Such inflammatory activity should resolve once the threat is overcome, becoming temporarily restricted and self-limiting to maintain homeostasis [9,10]. However, failure of immune resolution or continued exposure to environmental and biological factors that promote the activation of the inflammatory response can lead to a chronic inflammatory process. This results in the presence of immune cells such as lymphocytes, macrophages and plasma cells in the tissue for long periods of time, as well as of proinflammatory cytokines, chemokines and other proinflammatory molecules [11,12]. Although this condition recognized as low-grade inflammation has minimal or no clinical manifestations, the prolonged inflammatory response can cause consequences for tissue health, which can develop into tissue fibrosis and possible loss of function [13].

The presence of low-grade inflammation disrupts the homeostatic balance, altering the crosstalk between immune and metabolic responses and promoting chronic metabolic inflammation. This so-called “metainflammation” is primarily caused by metabolic and nutrient excess and triggers immune cell infiltration and the secretion of inflammatory cytokines into the tissue environment, which may inhibit glucose uptake or alter lipid metabolism [2,14]. As a result, chronic metabolic inflammation is particularly associated with an increased risk of noncommunicable diseases, such as cancer, diabetes and cardiovascular disease. An example is insulin resistance caused by chronic exposure to inflammatory biomarkers, which often lead to diabetes [15]. Low-grade inflammation plays an important role also in the development of cardiovascular diseases, due to its involvement in atheroprogression [16], and may favor the progression of different types of cancer by promoting cell proliferation, decreasing apoptosis and increasing angiogenesis and metastasis [17]. At present, it is not well-established which biomarkers can best represent low-grade inflammation, although among the most widely used in scientific studies are soluble mediators (chemokines and cytokines), acute-phase proteins (fibrinogen and C-Reactive Protein (CRP)) or blood cellular markers (granulocytes and total white blood cells) [18].

### Diet as a Risk Factor for Low-Grade Inflammation

**Figure 1:**
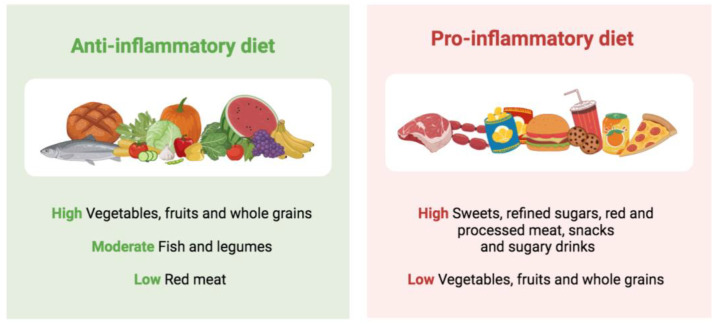
Dietary patterns and inflammation.

Among the environmental and lifestyle factors that can promote or intensify inflammation, increasing scientific evidence supports the role of diet. Potential nutritional compounds influencing inflammation processes include macro- and micronutrients, bioactive molecules such as polyphenols and specific food components [19]. Overall, plant-based dietary patterns with a high consumption of vegetables, fruits and whole grains, a moderate consumption of legumes and fish and a low consumption of red meat have been associated with a greater anti-inflammatory potential (Figure 1). These include several traditional healthy diets, such as the Mediterranean or the Nordic diet, which are usually based on minimally processed or unprocessed foods [20,21]. A meta-analysis that evaluated a total of 2300 subjects from 17 clinical trials showed that greater adherence to the Mediterranean diet was associated with lower levels of inflammatory biomarkers, particularly CRP and interleukin-6 (IL-6) [22]. These findings were confirmed in a recent meta-analysis assessing the effect of multiple dietary patterns on inflammatory biomarkers [23]. The authors concluded that the Mediterranean diet appeared as the dietary pattern with the most significant reductions in inflammatory biomarkers, including IL-6 and CRP [23]. Similar results were observed for the Nordic diet, with a review of intervention and observational studies revealing its beneficial influence on low-grade inflammation amelioration [24].

A growing number of studies show that the protective effects of these dietary patterns against inflammation are related to the dietary pattern as a whole, not just to its individual components [19]. All these dietary models share the presence of whole grains, fiber, vegetables, fruits, fish, polyunsaturated fatty acids (PUFAs), particularly marine *n*-3 PUFAs, vitamin C, vitamin E and carotenoids. In contrast, dietary factors that promote inflammation are oxidized lipids, saturated fatty acids (SFAs) and trans fatty acids, which are present at high levels in Western dietary patterns. Unfortunately, in recent years, the increased availability and variety of foods has led to a change in traditional dietary patterns, favoring a nutritional transition and a globalization of the diet towards a Western dietary pattern [25]. This dietary pattern, characterized by a high caloric intake and a high consumption of sweets, refined cereals, red and processed meats, snacks and sugary drinks, has been associated with an increased pro-inflammatory potential and higher levels of CRP and IL-6 [26].

To further investigate the role of diet in modulating inflammation, several literature-based indices have been developed. The energy-adjusted dietary inflammatory index (E-DII) analyzes the potential effect of 45 dietary elements on 6 inflammatory markers, both pro-inflammatory (IL-1b, IL-6, tumor necrosis factor (TNF)-α and CRP) and anti-inflammatory (IL-4, IL-10). The Empirical Diet Inflammatory Pattern (EDIP) is based on food group consumption and divides the dietary intake into nine inflammatory and nine anti-inflammatory food groups according to their impact on the CRP, IL-6 and TNF-αR2 biomarkers of inflammation [27]. Using these indices, many studies have assessed the potential inflammatory effect of diet on the health status. Recently, an umbrella review was conducted on DII and human health [28]. Umbrella reviews are overviews of systematic reviews and meta-analyses that provide a comprehensive and systematic evaluation of the scientific literature available for a specific research topic and offer the possibility to understand the strength of the evidence and the extent of potential biases [29]. In their umbrella review [28], authors found strong evidence supporting the relationship between a high dietary inflammatory index and an increased risk of myocardial infarction. They also found highly suggestive evidence for increased risk of cancer, in particular oral, respiratory, pancreatic and colorectal cancer, and all-cause mortality [28]. As for EDIP, several observational studies have associated a higher score with increased fasting blood sugar and decreased high-density lipoprotein (HDL) cholesterol levels, as well as with an increased risk of weight gain, metabolic syndrome, nonalcoholic fatty liver disease, heart failure and depression [30,31,32,33,34,35,36].

## 3. Ultra-Processed Foods (UPF)

One of the cornerstones of the Western diet are UPF, widely available and increasingly consumed in the contemporary society [4,37]. The possible role of UPF in the nutrition–health relationship was first highlighted by Monteiro et al. in 2009, with the introduction of the NOVA classification [38]. NOVA is a system that groups foods according to the nature, extent and purpose of the industrial processes they undergo, rather than in terms of the nutrients they contain [38]. In this classification, foods are assigned to one of four groups: Group 1 contains unprocessed or minimally processed foods, i.e., the edible parts of plants or animals taken directly from nature or minimally modified/preserved; Group 2 contains processed culinary ingredients, such as salt, sugar, oil or starch, produced from Group 1 foods; Group 3 contains processed foods such as canned vegetables or freshly baked bread, produced by combining Group 1 and Group 2 foods; Group 4 contains UPFs, defined as “formulations of ingredients, mostly of exclusive industrial use, that have little or none of the food intact and are typically created by a range of industrial techniques and processes” [38]. UPFs are identified by a long list of ingredients, are ready-to-eat, highly palatable, and usually inexpensive. The most commonly consumed UPFs include soft and sweetened beverages, processed bread, refined breakfast cereals, confectionery products, pre-packaged sauces, ready-to-heat meals and processed meats products [39]. Possible mechanisms behind their link with the health status may involve both their nutritional composition and “processing”. Indeed, in terms of nutritional composition, UPF are typically nutritionally unbalanced due to their ingredients [40]. Most UPF are energy-dense products high in added sugars, saturated and trans fatty acids and sodium and low in protein, fiber and certain micronutrients including potassium, magnesium, vitamin C, vitamin D, zinc, phosphorus, vitamin B12 and niacin [40].

UPF are also characterized by the presence of non-nutritive components, such as additives and chemicals. Additives are frequently added to make the final product more palatable, with better sensory qualities and longer shelf life. Commonly used additives in the manufacture of UPF include flavorings, emulsifiers and sweeteners such as aspartame, cyclamate or stevia-derived compounds [41]. As to the supposed presence of harmful chemicals in UPF, it has been suggested that they may derive from the processing or packaging of these products [42]. Processing could also alter the physical properties of food products, leading to a higher glycemic load and a reduced gut–brain satiety signaling, both responsible for overconsumption [43].

According to previous studies, all these aspects could explain the reason why the incidence of several chronic noncommunicable diseases is increasing along with UPF consumption [41]. Among adults, multiple meta-analyses found that a higher UPF consumption is significantly associated with an increased risk of overweight and obesity, metabolic syndrome, hypertension, diabetes and cardiovascular disease [6,44,45,46,47]. A higher UPF consumption has also been associated with a higher risk of cancer, particularly breast cancer [6,48], anxiety and depression [49] and all-cause mortality [50,51]. In children and adolescents, significant relationships were found with overweight and obesity [25,52].

## 4. UPF and Low-Grade Inflammation

**Figure 2:**
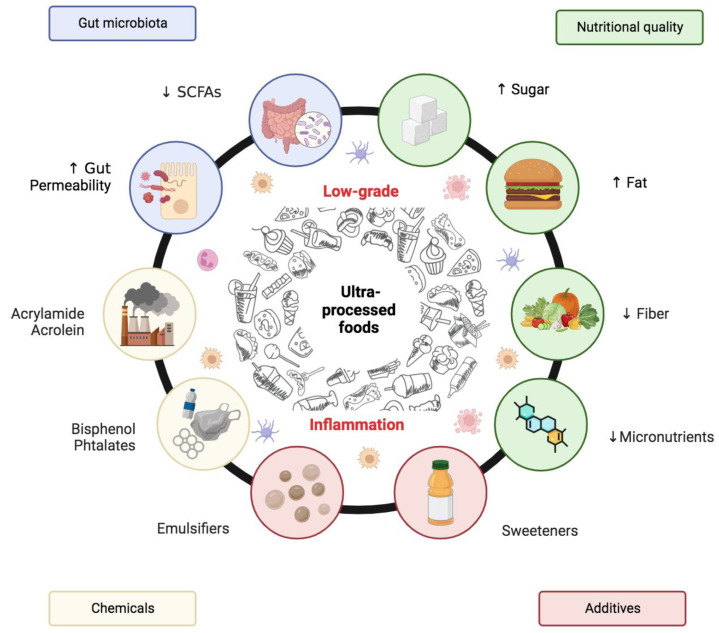
Possible mechanisms explaining the relationship between UPF and low-grade inflammation. ↑ increased; ↓ reduced.

The number of human studies investigating whether the consumption of UPF could promote low-grade inflammation, so favoring the development of noncommunicable diseases, is still limited. The available studies have focused mainly on two aspects: how excessive UPF consumption may affect the presence of biomarkers of inflammation, and how the nutritional composition or non-nutritional components of UPF may influence the development of chronic inflammation and gut dysbiosis, previously correlated with a pro-inflammatory state (Figure 2).

The vast majority of studies that have examined the relationship between UPF consumption and inflammation are observational, either cross-sectional or cohort studies (Table 1), with only one clinical trial currently available [53].

CRP is the most investigated inflammatory biomarker to date in relation to UPF consumption. In the only available clinical trial, subjects assigned to a diet based on unprocessed foods showed a significant reduction in hs-CRP levels, while subjects on a diet rich in UPF did not report significant changes [53]. The authors suggested that these results might indicate that the subjects were already regularly consuming a large amount of UPF, as already observed in the US population [53]. As for data from observational studies, they are not consistent and suggest that the relationship may depend on gender and body mass index (BMI). For example, in the ELSA-Brasil study, a significant association between high UPF consumption and higher CRP levels was found in women, but the association lost its significance when adjusting for BMI [54]. Similarly, in the Melbourne Collaborative Cohort Study, the association between high UPF consumption and CRP levels remained significant only in men, after adjustment for BMI [55]. In adolescents, Martins et al. found that subjects consuming more UPF in their diet had higher CRP and IL-8 values, but the association was significant only for IL-8 [56]. Other biomarkers studied to a lesser extent are some proinflammatory cytokines such as IL-6. Dos Santos et al. investigated the possible relationship between UPF consumption and IL-6 concentrations in two cohorts, showing an association only in women in the Portuguese cohort and only in men in the Brazilian cohort [57]. The conclusion was that the UPF intake could be associated with higher IL-6 levels, although the relation was not explained by adiposity [57].

As to the E-DII score, a cross-sectional study in Brazil found a direct relationship between a higher dietary energy intake from UPF and a higher rate of dietary inflammation in pregnant women [58]. Similar findings were obtained in the Italian cohort Moli-Sani, where a higher consumption of UPF was related to a higher pro-inflammatory potential of the adults’ diet [59]. In this cohort, further analyses were performed using the low-grade inflammation (INFLA)-Score, which allows the assessment of the possible intensity of low-grade inflammation through the effects of biomarkers of inflammation (platelets, white blood cell (WBC), CRP and granulocyte-to-lymphocyte ratio), obtaining the same association [59].

## 5. Possible Mechanisms Explaining the Relationship between UPF and Low-Grade Inflammation

### 5.1. Nutritional Aspects

UPF consumption could contribute to an inflammatory state through several mechanisms. First, it could be the high intake of sugars, salt, saturated fats and trans fatty acids typical of a UPF-rich diet that directly promotes the development of chronic inflammation [61]. When high intakes of these nutrients and their possible relationship to the modulation of inflammation are considered individually, the results to date are mixed. UPF are usually high in simple sugars, in the form of either sucrose or a high-fructose syrup, so they tend to be foods that raise the blood glucose markedly and rapidly, i.e., with a high glycemic index/glycemic load [62]. This postprandial increase in the glucose levels in turn causes an increase in insulin levels, which promotes a proinflammatory state [63]. Although these mechanisms appear to play an important role in diet and the promotion of low-grade inflammation, intervention studies are not very clear in this regard. In the TOSCA.IT study, an association was found between the intake of added sugars ≥10% of the daily energy intake and increased CRP levels in adults with diabetes [64]. Other observational studies associated a higher consumption of sugar-sweetened beverages with increased levels of CRP and IL-6 in adults and children [65,66,67]. Regarding the glycemic response, although an intervention study found a positive association between glycemic load and plasma hs-CRP in healthy middle-aged women [68], a recent meta-analysis including 28 randomized controlled trials found no association between the glycemic index and different markers of inflammation in adults [69].

UPF also have a high salt content, contributing to a high sodium intake. Several cross-sectional studies associated a higher salt intake with higher CRP levels in adults and elderly people [70,71], although this association was not found in adolescents [72]. A recent meta-analysis also found no associations between dietary sodium level and markers of inflammation, although it should be noted that the researchers pointed out that their findings were likely due to methodological errors [73].

As for the fat content of UPF, their inflammatory potential derives not only from a higher consumed quantity with respect to other foods, but also from a poorer quality. In fact, trans fatty acids resulting from the industrial process are associated with a higher presence of low-grade inflammation. Specifically, they have been related to higher levels of hs-CRP, IL-6 and TNF-α [74,75,76]. Diets with a high processed-food content have also been associated with a higher intake of omega-6 fatty acids, resulting in a higher omega-6/omega-3 ratio and the potential promotion of low-grade inflammation [77].

Finally, consuming large amounts of UPF sometimes results in the replacement of foods that are the basis of a healthy and balanced diet. Examples are fruits and vegetables, which are correlated with an anti-inflammatory effect thanks to the presence of numerous phytocompounds [78,79]. Recent studies clearly show how people consuming more UPF have a lower intake of fruit and vegetables [80] and consequently ingest less substances with an anti-inflammatory effect. A low fruit and vegetable consumption also results in a low dietary fiber intake. In the E-DIITM, fiber is considered one of the factors that reduce diet-related inflammation. In previous studies, an adequate fiber intake was shown to be important in maintaining low CRP levels and in maintaining homeostasis of the gut microbiota [81]. A high UPF consumption can also lead to deficiencies of micronutrients considered to be anti-inflammatory factors in the diet, such as magnesium, vitamin C, vitamin D, zinc and niacin [82].

### 5.2. Non-Nutritional Aspects

Results from an Italian cohort study suggested that only part of the proinflammatory effect of a high UPF consumption can be directly attributed to the nutritional components of the diet, while the rest could be attributed to non-nutritional factors that may promote low-grade inflammation [59]. One of the non-nutritional factors present in UPF are additives, which are added to mimic or intensify the sensory qualities of foods [83]. Among the most studied are sweeteners, especially non-caloric ones such as acesulfame potassium, sucralose or aspartame, due to their widespread use in soft drinks to provide a sweet taste without the energy value of sugars [84]. Recently, there has also been growing interest in the harmful effect of emulsifiers used to improve the shelf life and texture of food products. Although scientific evidence to date is limited, animal and in vitro studies suggest that sweeteners and emulsifiers may contribute to the inflammatory cascade [85,86,87]. One of the hypothesized mechanisms is the modulation of the microbiota, but data are inconsistent, and further studies are needed to investigate these mechanisms [88,89]. It has also been hypothesized that the non-caloric sweeteners’ harmful effect might be due to an acute metabolic response [90]. However, data from two recent meta-analyses do not support this hypothesis, as they found no association between the consumption of non-caloric sweetened beverages and an increased insulinemic effect or acute glycemic response [91,92].

Non-nutrient components such as bisphenol or phthalates may also be present in UPF due to the migration of chemical substances that are part of food packaging. In fact, several cross-sectional studies reported higher levels of both substances in the urine of people with a high UPF consumption [42,93,94,95,96]. Because of their structure, bisphenol and phthalates can disrupt various aspects of the hormonal action and are therefore called endocrine disruptors. They can interfere with the synthesis, secretion, transport, signaling and metabolism of hormones; therefore, they have been associated with adverse health consequences, including the development of diseases such as obesity, diabetes and cardiovascular disease [97,98].

A recent meta-analysis investigating the role of different endocrine disruptors on the inflammatory response showed that increased exposure to Bisphenol A (BPA) is significantly associated with higher levels of IL-6 and CRP, while increased exposure to phthalates is associated with higher levels of CRP, IL-6 and IL-10 [99]. Although the adverse effects of BPA have led to various restrictions on its use, the analogs that replaced it appear to have similar effects [100]. On the other hand, UPF may contain chemicals derived from food processing, especially due to the heat treatment to which food is subjected. One example is acrylamide as a result of the Maillard reaction between amino acids and sugars, exposure to which in adults has been associated with an increased presence of biomarkers of inflammation such as CRP or Mean Platelet Volume (MPV) [101]. Another chemical instead derived from lipid oxidation is acrolein, high exposure to which has been associated with a higher concentration of Hs-CRP in adults in the United States [102] and of CRP in adults in China [103].

### 5.3. Gut Microbiota Modulation

The human gut microbiota is a dynamic and complex network composed of hundreds of thousands of microorganisms, including bacteria, fungi, archaea, viruses and protozoa [104]. When in its normal state of homeostasis, the gut microbiota plays a key role in host health through the immune system function and protection against pathogens. However, when the gut microbiota is altered compared to the community found in healthy individuals, gut dysbiosis occurs [84]. This dysbiosis is associated with a high degree of inflammation, caused by a lower presence of short- chain-fatty-acids-(SCFAs)-producing bacteria, and increased permeability of the gut [105]. Both diet quality and the presence of the additives previously described may influence intestinal dysbiosis, offering a possible explanation for the mechanism linking an increased consumption of UPF with the presence of low-grade inflammation.

In fact, it has been suggested that a diet rich in fiber can decrease the systemic inflammatory response by improving the intestinal barrier function and modulating the intestinal microbiota [81]. This is because dietary fiber is essential for the formation of SCFAs, which are thought to play a key role in neuroimmunoendocrine regulation [106]. In fact, SCFAs are associated with a lower concentration of CRP and plasma lipopolysaccharide, an endotoxin used as a marker to assess intestinal permeability linked to increased low-grade inflammation [107,108,109,110]. In contrast, Western diets with a high fat content have been associated with increased intestinal permeability due to a greater presence of lipopolysaccharides in humans and mice [111,112]. Similar results were observed in mice fed a diet rich in refined sugar, also associated with an atypical composition of the intestinal microbiota [113]. In a cross-sectional study conducted in the U.S.A., the increased consumption of highly processed food was associated with intestinal permeability biomarkers [114]. Also in a study conducted in Italy, intestinal permeability tended to increase in subjects with low adherence to the Mediterranean diet, who also reported a high intake of food high in fat and sugar, referred to as junk food [115]. Finally, a French study involving 862 healthy adults found that the regular consumption of foods such as soft drinks, fatty sweet products, fried foods, processed meats, ready-to-eat meals, cheese and desserts, most of them recognized as UPF, was associated with reduced bacterial diversity, indicating an altered microbiota composition [116]. In contrast, the PREDIMED-PLUS study in older adults found no such association and suggested that perhaps the contradictory results with the previous study were due to the lower UPF consumption of the studied population [117].

Several studies have also highlighted additives as possible factors affecting the microbiota. Studies in murine models suggested different mechanisms through which emulsifying additives could contribute to intestinal dysbiosis, increasing intestinal permeability and promoting a proinflammatory state [89,118]. However, these studies remain limited, and the results in humans are contrasting. For example, a double-blind controlled study comparing seven adults on an emulsifier-rich diet to nine adults on an emulsifier-free diet observed changes in the gut microbiome and metabolome that may be related to chronic inflammatory diseases [119]. In contrast, a cross-sectional study involving 588 adults found no association with biomarkers related to increased intestinal permeability, although it found an association with increased levels of systemic inflammation [114]. Similarly, studies in murine models suggested that artificial sweeteners can alter the intestinal microbiota, favoring the enrichment of proinflammatory bacteria that promote the formation of endotoxins such as lipopolysaccharides [85,86,120]. However, the results to date are inconsistent, and further research will be needed to investigate these mechanisms.

## 6. Conclusions and Future Perspectives

Low-grade inflammation plays a pivotal role in the pathogenesis of noncommunicable diseases, which are becoming increasingly prevalent worldwide. In recent years, diet has been highlighted as one of the main risk factors for these diseases, together with the increased consumption of UPF, which through different mechanisms, may contribute to promote a proinflammatory state. Although the evidence on the association between UPF consumption and inflammation is still limited and, in some cases, the results are discordant, considering the potential impact of their excessive consumption on the health status, as well as their potential role in favoring the presence of chronic inflammation, public policies that limit their consumption are required. These public policies should also include the promotion of traditional diets based on unprocessed or minimally processed foods, in order to modulate low-grade inflammation and improve people’s health status. Future human research evaluating clusters of inflammation markers instead of individual biomarkers may help to better understand the mechanism involved in the modulation of low-grade inflammation by a high consumption of UPF. This information could also be useful in establishing policies that promote the reformulation of UPF to minimize their adverse health effects.

## Figures and Tables

**Table 1:** Observational studies assessing the relationship between UPF consumption and inflammatory biomarkers.

Author, Year	Study Design	Country	Participants, n	Gender	Age	Study Population	Outcome	Main Results
Lopes et al., 2019 [54]	Cross-sectional analysis of Longitudinal Study of Adult Health (ELSA-Brasil) baseline cohort	Brazil	8468	M/F	35–74	General population	CRP	A higher tertile of UPF intake was associated with a 14% increase in CRP levels only among women. Significance was lost when adjusting for BMI.
Lane et al., 2022 [55]	Cross-sectional analysis of Melbourne Collaborative Cohort	Australia	2018	M/F	57 ± 9	General population	hs-CRP	A 100 g increase in UPF consumption was associated with a 4% increase in hs-CRP concentration, independently of BMI.
Martins et al., 2022 [56]	Cross-sectional	Brazil	391	M/F	17–18	General population	Leptin, IL-6, IL-8, CRP TNF- α	The highest tertiles of UPF intake showed higher levels of CRP and serum leptin and a 79% increase in IL-8 levels. No association was found for IL-6 and TNF-α
Silva Dos Santos et al., 2022 [57]	Cross-sectional analysis of EPITeen Cohort and Pelotas Birth Cohort	Brazil, Portugal	3412	M/F	27–30	General population	IL-6	A positive association between levels of IL-6 and UPF intake was found among females from the Portugal cohort and males from the Brazil cohort.
Kesley et al., 2022 [58]	Cross-sectional analysis of Norwegian Mother, Father and Child Cohort	Norway	2984	F	30 ± 4	Pregnant women	CRP	An increase UPF intake was associated with a 5.4% increase in CRP levels, even after adjustment for pre-pregnancy BMI
Mignogna et al., 2022 [59]	Cross-sectional analysis of Moli-sani cohort	Italy	21,315	M/F	55 ± 3	General population	INFLA-score E-DII score	INFLA-score was associated with higher E-DII score and UPF intake. When adjusting for E-DII, the association of UPF with the INFLA-score was mitigated by 32.6%
Silva et al., 2019 [60]	Cross-sectional	Brazil	784	F	28 ± 5	Pregnant women	E-DII score	E-DII score was positively associated with consumption of UPF when adjusting for covariates including pre-pregnancy BMI

UPF: ultra-processed foods; CRP: C-reactive protein; BMI: body mass index; hs-CRP: high-sensitivity C-reactive protein; IL: interleukin; TNF: tumor necrosis factor; INFLA: low-grade inflammation; E-DII: energy-adjusted dietary inflammatory index.

## Data Availability

Additional data are available from the corresponding author on reasonable request.
